# The explorer's edge: Racial-ethnic identity exploration confers early adolescents of color with protection against racial-ethnic discrimination in a co-sibling control study

**DOI:** 10.1093/chidev/aacaf048

**Published:** 2026-02-11

**Authors:** Juan Del Toro, Warren Christopher L Aguiling, Junqiang Dai, Charissa S L Cheah, Qi Huang, Mohammad Hashim, Binhao Wu

**Affiliations:** Department of Psychology, University of Minnesota-Twin Cities, Minneapolis, MN, United States; Department of Psychology, University of Maryland-Baltimore County, Baltimore, MD, United States; Department of Psychology, Georgia State University, Atlanta, GA, United States; Department of Psychology, University of Maryland-Baltimore County, Baltimore, MD, United States; Department of Psychology, University of Minnesota-Twin Cities, Minneapolis, MN, United States; Department of Psychology, University of Minnesota-Twin Cities, Minneapolis, MN, United States; Department of Psychology, University of Minnesota-Twin Cities, Minneapolis, MN, United States

**Keywords:** racial-ethnic identity development, racial-ethnic discrimination, psychopathology symptoms

## Abstract

Racial-ethnic identity development may help adolescents cope with racial-ethnic discrimination. Exploration reflects efforts to understand one's racial-ethnic background, whereas commitment represents a sense of connection to one's racial-ethnic group. The present study investigated whether these identity components moderated associations between discrimination and psychopathology symptoms one year later among 1,184 adolescents of color (ages: 11–12; 52% female, 48% male; 35% Black, 36% Latino, 3% Asian, 26% Other youth of color; Waves 3–4: 2019–2022) nested within 656 families. In sibling fixed-effect models, adolescents reporting greater racial-ethnic identity exploration than their siblings showed weaker associations between discrimination and psychopathology symptoms. Racial-ethnic identity commitment did not moderate these associations. Findings underscore the developmental significance of identity exploration as a protective process during early adolescence.

Early adolescence is a period of excitement when youth of color begin to wander and explore who they are and who they want to be (Fuligni & Galván, 2022). Relative to children, early adolescents have developed greater cognitive abilities, social awareness, and independence (Casey et al., 2025; Hashim, 2024), enabling them to ask more complex questions about their racial-ethnic identities (Meeus, 2017). Racial-ethnic identity is the part of one's social identity that is based on membership in one or more racial-ethnic groups (Umaña-Taylor & Rivas-Drake, 2021; Verkuyten, 2016). Racial-ethnic identity includes the content, meaning, and significance of one's race-ethnicity (i.e., content models; Sellers et al., 1997) and the process through which racial-ethnic identity develops (i.e., development models; Hughes et al., 2017; Umaña-Taylor et al., 2014). Relative to identity content, identity development includes behavioral aspects (e.g., asking questions, formulating strong bonds with same racial-ethnic peers) that can be enacted in day-to-day life (Ashmore et al., 2004; Cross et al., 2017). These behavioral components of racial-ethnic identity are especially consequential during early adolescence, when most youth begin to formulate racial-ethnic identities characterized by a sense of belonging to their group (Meeus, 2011). Adolescents who engage in racial-ethnic identity development demonstrate better psychological well-being (Elmore et al., 2012; Smith & Silva, 2011), academic achievement (Del Toro & Wang, 2021; Miller-Cotto & Byrnes, 2016), and physical health (Rivas-Drake et al., 2014).

Racial-ethnic identity development is a dynamic process that is reflected in its multidimensional components, each of which may offer adolescents protection against racial-ethnic discrimination. Racial-ethnic identity development is mainly comprised of two dimensions: Racial-ethnic identity exploration and racial-ethnic identity commitment (Phinney & Ong, 2007). Racial-ethnic identity exploration reflects the practices youth engage in to explore and learn more about their racial-ethnic identities, whereas commitment reflects youth's sense of belonging with and connection to their racial-ethnic identities. The openness to new perspectives as part of racial-ethnic identity exploration may enable adolescents to have conversations about discrimination with close others, who may offer adolescents support and dialogue to reframe their negative experiences (McLean & Pasupathi, 2012). In addition, feeling belonging and connected to others as part of racial-ethnic identity commitment may preemptively offer adolescents a close-knit community who can relate to youth and represent a sounding board when youth vent about their discrimination experiences (Del Toro et al., 2024a). Altogether, these multiple components of racial-ethnic identity development may help youth cope with discrimination; yet, most studies examining this particular role of racial-ethnic identity development have been cross-sectional in nature (Yip et al., 2019). Therefore, the lack of longitudinal studies using repeated assessments to address the developmental and temporal complexity of racial-ethnic discrimination, racial-ethnic identity development, and adolescents’ adjustment outcomes is a gap that warrants more attention from the field.

One design that may offer enhanced internal validity in the study of the risk tied to and resilience against racial-ethnic discrimination is the co-sibling control design. When adolescents and their siblings are reared together by the same family, both shared and unshared environmental factors shape their development. Shared environmental factors are those that make siblings’ experiences more similar, such as household (e.g., parental education, income, marital status), neighborhood (e.g., proportion of residents within a census tract living below the poverty rate), and school characteristics (e.g., a school's physical architecture and cultural dynamics). In addition to growing up in the same household and neighborhood as well as attending the same school, adolescents formulate different peer groups, enroll in separate classrooms, and have unique relationships with their parents (McGuire & Segal, 2013; Segal, 2019). These factors are examples of unshared environmental factors, which are those that make siblings more distinct. Within this framework, racial-ethnic discrimination can reflect both shared and unshared environmental influences. Racial-ethnic discrimination is defined as the behavioral manifestation of racism and constitutes unfair and prejudicial treatment directed at individuals based on their race-ethnicity (Williams et al., 2019). For instance, siblings may share direct and vicarious experiences of discrimination when their family or community is targeted yet also encounter unique discriminatory events in their own peer, school, or social contexts. By holding constant shared environmental factors that commonly confound associations with racial-ethnic discrimination (e.g., household and neighborhood indicators of socioeconomic status; Del Toro et al., 2022; Laub, 2014), co-sibling designs can help disentangle the unique and common effects of discrimination on adolescents’ adjustment outcomes.

To contribute to the dialogue regarding the protective role of racial-ethnic identity development, the present study leveraged a racially-ethnically diverse sample of adolescents nested in sibling relationships from the Adolescent Brain Cognitive Development (ABCD) study. These nested data strengthen internal validity relative to cross-sectional approaches by adjusting for self-selection bias and unmeasured environmental factors shared between siblings (Rutter, 2007). Using this co-sibling control design, we tested whether adolescents who perceived more racial-ethnic discrimination than their siblings also reported more psychopathology symptoms one year later and whether adolescents who reported greater levels of racial-ethnic identity development components than their siblings showed more resilience, or recovery from harms associated with discrimination (Del Toro et al., 2024c; Masten, 2024).

## Conceptual framework: racial-ethnic discrimination and psychopathology symptoms

The Phenomenological Variant of Ecological Systems Theory (PVEST) provides a framework for understanding how adolescents interpret and respond to racial-ethnic discrimination (Cunningham et al., 2023; Spencer, 1995). Central to PVEST is the process of meaning making, where individuals actively construct understandings of their lived experiences. One instance in which meaning making is activated is when adolescents encounter racial-ethnic discrimination, which is an intermediate experience of stress derived from stereotypes and biases (Spencer et al., 1997). These stereotypes and biases, often linked to social characteristics like race-ethnicity, directly shape how environmental figures (e.g., peers, teachers, police) perceive and treat youth of color, and these interactions may include racial-ethnic discrimination (Spencer et al., 1997). The meaning making derived from experiences with discrimination may become internalized over time among youth of color. This internalization can reinforce maladaptive stress responses, influencing psychopathology symptoms (Cunningham et al., 2023; Spencer, 1995).

In line with the PVEST (Cunningham et al., 2023; Spencer, 1995), individuals’ social characteristics, such as their race-ethnicity, can increase their exposure to racial-ethnic discrimination. Adolescents of color experience racial-ethnic discrimination of various kinds from diverse perpetrators, ranging from peer victimization (Seaton et al., 2022), severe discipline for minor violations (Del Toro & Wang, 2022), and stop-and-frisks by the police (Del Toro et al., 2019; Hughes et al., 2016). By early adolescence, as many as one-in-four youth of color have perceived racial-ethnic discrimination at least once in the past 6 months (Del Toro et al., 2024d) and in an unspecified timeframe (Del Toro et al., 2024a).

Given the widespread prevalence of racial-ethnic discrimination among adolescents, youth are actively interpreting and internalizing these experiences. The PVEST framework posits that racial-ethnic discrimination can serve as a risk factor that contributes to long-term psychological distress, particularly through reactive coping methods (Cunningham et al., 2023; Spencer, 1995). Reactive coping methods are problem-solving strategies, ranging from adaptive to maladaptive (Spencer et al., 1997). Over time, maladaptive responses can be internalized and shape youth psychopathology trajectories (Spencer, 1995; Spencer et al., 1997). When youth repeatedly encounter discrimination, such as being perceived as less intelligent and more threatening (Bai & Zhao, 2024; Goff et al., 2014), they make sense of these experiences within their broader social and cultural contexts. Through meaning making processes, these societal messages can become embedded in their self-concepts, shaping how youth view themselves and their place in the world. This internalization can contribute to lower self-esteem, heightened stress responses, and depressive symptoms (Benner et al., 2018, 2022). Over time, these experiences can reinforce maladaptive coping strategies, such as avoidance or disengagement, potentially exacerbating psychopathology symptoms among racially-ethnically marginalized youth (Cunningham et al., 2023; Spencer, 1995).

Empirical research further supports the role of racial-ethnic discrimination as a contextual stressor that shapes adolescents’ psychopathology symptoms. Multiple meta-analyses have shown that racial-ethnic discrimination is associated with increased depressive symptoms, lower self-esteem, heightened psychological distress, and greater engagement in risk-taking behaviors (Benner et al., 2018, 2022). For example, in a six-year longitudinal study, racial-ethnic discrimination during early adolescence was associated with increased depressive symptoms, lower self-esteem, and greater behavioral problems during middle and late adolescence (Hughes et al., 2016). Even studies using daily diary designs–which make within-person comparisons and account for unmeasured confounds that bias between-person analyses–found that youth report more negative affect, depressive symptoms, and anxiety on days when they experience more discrimination compared to days with little or no discrimination (Del Toro et al., 2024b; Del Toro & Wang, 2023; Wang et al., 2025). Because the harmful effects of discrimination on psychopathology symptoms are unequivocal, it is essential to further explore the complexities of this relation among early adolescents from racially-ethnically diverse backgrounds.

## The moderating role of racial-ethnic identity development

According to the phenomenological component of the PVEST, youth develop reactive coping methods following racial-ethnic discrimination through their emergent identities (Cunningham et al., 2023; Spencer et al., 1997). During adolescence, ego-identity development, including racial-ethnic identity development, is a central task (Erikson, 1968; Spencer, 1995). Racial-ethnic identity development originated from ego identity formation (Erikson, 1968) and emerged from prior conceptualizations of identity development that reflected a teleological process with an end state (Marcia, 1966; Phinney, 1989). This process entailed an *unexamined* stage, in which the meaning of one's group membership is unexplored, followed by sequential stages of exploration and achievement (Phinney, 1989). Identity achievement ideally resulted in commitment to one's race-ethnicity and active participation in identity-relevant contexts (Phinney, 1989). Over time, scholars found that identity development components reformulate throughout the life cycle (Phinney & Ong, 2007) with day-to-day fluctuations (Cross et al., 2017; Del Toro et al., 2024a). These advancements in the racial-ethnic identity development literature led to the distinction of two but related components of racial-ethnic identity development, including racial-ethnic identity exploration and commitment (Phinney & Ong, 2007).

The PVEST emphasizes that reactive coping methods are context-dependent corrective problem-solving strategies that are linked to youth's emergent identities, shaping their responses and adaptations to their environments (Spencer et al., 1997). Therefore, components of racial-ethnic identity development as reactive coping methods to the broader context of racialization may play a critical role in conferring adolescents of color with protection against acts of racial-ethnic discrimination (Yip, 2018; Yip et al., 2019). Following discrimination, adolescents who explore their racial-ethnic identities may demonstrate greater adaptability, as their evolving sense of self remains more flexible and open to new perspectives (McLean & Pasupathi, 2012). This broadened perspective can enable youth to reframe their experiences of racial-ethnic discrimination not solely as negative incidents but as moments that foster self-reflection and personal growth (Pasupathi et al., 2012). In addition, the strengthened sense of belonging with racial-ethnic peers as part of racial-ethnic identity commitment may imbue discriminated youth with solidarity and community that could disrupt youth's internalization of negative stereotypes (Torres et al., 2011) and enable them to more readily focus on the positive aspects of their racial-ethnic membership following discrimination experiences (Shelton et al., 2005).

Despite theoretical support for the protective roles of racial-ethnic identity exploration and commitment, empirical findings have been less consistent (Yip, 2018; Yip et al., 2019). These trends have been supported by the literature as a comprehensive meta-analysis of 51 studies among 18,545 participants found that racial-ethnic identity exploration exacerbated the harmful effects of racial-ethnic discrimination on individuals’ adjustment outcomes and racial-ethnic identity commitment weakened the deleterious impacts of racial-ethnic discrimination on such adjustment outcomes (Yip et al., 2019). However, 48 of the 51 studies in the meta-analysis were cross-sectional, which include limitations, such as obscured direction of effects between key constructs and self-selection bias (Maxwell et al., 2011). Moreover, cross-sectional studies rely on between-person comparisons that are susceptible to unmeasured confounding, particularly from differences between participants’ environments, such as neighborhood characteristics (e.g., poverty rate of census tract, crime rates) that are highly correlated with racial-ethnic stratification (Laub, 2014). As a result, whether racial-ethnic identity development would continue to confer youth with protection against discrimination once additional confounds are addressed with more rigorous designs remains an empirical question.

## The utility of co-sibling control designs

One quasi-experimental design underutilized in research on racial-ethnic identity is the co-sibling control design (Del Toro et al., 2025a; Rea-Sandin et al., 2023; Rutter, 2007). Full siblings and dizygotic twins, on average, share 50% of their genetic architecture, making it likely that they will have different sexes, skin tones, and other physical appearances (Laffey-Ardley & Thorpe, 2006). This phenotypic variation between siblings in the same family can influence how they may be perceived and treated by others (Marteleto & Dondero, 2016). Even though monozygotic twins are, on average, genetically identical, each one will have a unique experience within their shared social ecology that will shape their perceptions and worldviews. Regardless of siblings’ zygosity, siblings differ in peer groups, school experiences, and engagement with various social activities (Eriksson et al., 2017; Plomin & Daniels, 2011). Indeed, empirical studies have found that siblings within a family significantly vary from each other in their racial-ethnic discrimination experiences (Del Toro et al., 2024a) and psychopathology symptoms (Burt, 2014). Thus, there is untapped variation between siblings within the same family that can be fruitful in the study of racial-ethnic-related processes.

Specifically, the co-sibling control design leverages adolescents nested in families. This multilevel framework is similar to that of other hierarchical structures where adolescents are nested in classrooms and other social settings, which enable scholars to rule out biases introduced when settings have different characteristics (e.g., different teachers; Burt, 2014). Relative to other multilevel designs, however, where placement in a classroom or neighborhood is not random (e.g., school tracking, redlining), siblings born into the same family is random. This random nature of siblings born into the same family adjusts for selection bias (McGue et al., 2010; Rutter, 2007). In addition, comparing siblings reared together helps account for shared environmental factors, such as parental education and income, which are invariant across siblings (McGue et al., 2010; Rutter, 2007). Although siblings may experience contexts differently via unshared environmental factors (e.g., due to age, birth order, or differential treatment by teachers and parents; McGue et al., 2010; Rutter, 2007), the co-sibling design reduces confounds from stable between-family differences that typically bias between-person analyses. Moreover, this co-sibling control design strengthens the assessment of the PVEST's phenomenological component by focusing on a sibling's unique experience and perception of their environments while controlling for shared environmental factors relative to their other siblings.

Indeed, research has shown that co-sibling control studies have produced conflicting results from those derived from between-person comparisons, which may extend to the protection linked to racial-ethnic identity development. In a separate literature where co-sibling control studies are common, one study with monozygotic twin pairs who were discordant on body mass index (BMI) found that the twin with a higher BMI had a more unfavorable blood biomarker profile compared to the twin with a lower BMI, and this association was not evident in general population studies (van Dongen et al., 2015). In a separate study of twin pairs discordant for depression, distinct DNA methylation differences emerged between the affected and unaffected twins, and these epigenetic differences were not apparent in singleton (i.e., unrelated individuals) studies (Dempster et al., 2014). One of the few studies with a strong design that assessed racial-­ethnic identity development used a 14-day daily diary that focused on intra-individual or within-person comparisons (Budescu et al., 2025). In this study, late adolescents’ racial-ethnic identity commitment and, surprisingly, exploration weakened the within-person effect of racial-ethnic discrimination on adolescents’ depressive symptoms, and this protection did not extend to the between-person effect of racial-ethnic discrimination on depressive symptoms (Budescu et al., 2025). Therefore, designs that account for a broader range of confounds than cross-sectional studies may offer more robust and nuanced insights.

## The present study

The current study used a co-sibling control design to investigate whether early adolescents’ racial-ethnic identity exploration and commitment buffered the longitudinal impact of racial-ethnic discrimination on their psychopathology symptoms one year later. We posed the following two research questions (1) Did early adolescents who perceived more racial-ethnic discrimination than their siblings report more psychopathology symptoms than their siblings one year later and (2) did early adolescents’ greater racial-ethnic identity exploration or commitment relative to their siblings one year following racial-ethnic discrimination modify the longitudinal association between racial-ethnic discrimination and their psychopathology symptoms?

We drew from theory and existing empirical studies to formulate confirmatory hypotheses for our two key research questions. For our first research question and due to the psychosocial stress associated with perceiving unfair treatment (Benner et al., 2022; Spencer, 2006), we predicted that early adolescents who perceived more racial-ethnic discrimination than their siblings would report more psychopathology symptoms one year later. As for our second research question, to capture the potential reactive coping properties tied to racial-ethnic identity development (Spencer et al., 1997), we measured racial-ethnic identity development one wave (i.e., year) following racial-ethnic discrimination. After adjusting for shared environmental factors between siblings in the same family and due to the salience of one's race-ethnicity to their self-concept in adolescence (Phinney & Ong, 2007; Yip et al., 2019), we predicted that adolescents’ self-reported higher levels of racial-ethnic identity exploration and commitment relative to their siblings would each independently confer protection and weaken the longitudinal association between racial-ethnic discrimination and psychopathology symptoms.

To robustly test our hypotheses, we leveraged the ABCD study for two methodological reasons. First, the ABCD study tracked yearly assessments of youth's psychopathology symptoms. Longitudinal data are key to ruling out pre-existing differences in symptoms and for predicting residual changes in future symptoms that cannot be explained by prior symptom levels. Second, national data of racially-ethnically minoritized siblings is almost absent in the literature, situating the sibling sub-sample of the ABCD study as the only of its kind to test research questions about racial-ethnic discrimination and identity development among siblings of color. Recall that siblings offer a quasi-experimental design, as adolescents born into and raised by the same family enable scholars to account for selection bias and unobserved heterogeneity, enhancing causal inference (McGue et al., 2010; Rutter, 2007). Integrating longitudinal and co-sibling control methods positions our study to make one of the most rigorous tests of hypotheses about racial-ethnic discrimination and identity development among adolescents of color.

In addition to our core research questions and design, we ran a series of supplemental exploratory analyses to understand the boundaries of our inferences. First, the combination of racial-ethnic identity exploration and commitment levels may offer protection against discrimination (Yip et al., 2019). Thus, we tested whether combining both racial-ethnic identity exploration and commitment into a single composite score moderated the association between racial-ethnic discrimination and psychopathology symptoms. Second, the sibling sub-sample of the ABCD study included monozygotic twins, dizygotic twins, and full siblings. Monozygotic twins share 100% of their genes, and dizygotic twins and full siblings share 50% of their genes. With this variability in siblings’ genetic relatedness with one another, we tested the idea that resilience linked to racial-ethnic identity development may be a heritable trait by assessing the moderating role of siblings’ zygosity. Third, to justify that our results were attributable to the co-sibling control design, we tested our research questions among the singleton sub-sample of the ABCD study. Last, given the racial-ethnic and sex diversity in the ABCD study, we explored the degree to which our primary results differed by adolescents’ racial-ethnic background and sex.

## Method

The ABCD study included data from 4,050 siblings and their primary caregivers nested in 1,989 families. After we excluded 2,409 White youth and 457 youth of color who did not have data on key constructs (i.e., racial-ethnic discrimination and racial-ethnic identity development), our analytic sample was comprised of 1,184 early adolescents (ages: 11–12 years; 35% Black, 36% Latino, 3% Asian, 26% Other youth of color; 52% females, 48% males) nested across 656 families. Each family had up to three adolescents (94% two-sibling households, 6% three-sibling households). Of the Other youth of color, 91% were multi-racial-ethnic, 3% identified as Other, 2% identified as Pacific Islander, 2% identified as American Indian, and 2% did not self-identify with a racial-ethnic label.

### Procedure

The ABCD study involves a longitudinal sample of 11,868 early adolescents from a 21-site consortium (Garavan et al., 2018). These early adolescents included singletons, non-twin siblings, and twins. Singletons were primarily recruited through public, charter, and private schools. School selection was informed by gender, race-ethnicity, socioeconomic status, and urbanicity to ensure that the participants’ demographic characteristics were close to the general U.S. population (Barch et al., 2018). Across 21 sites, adolescents’ non-twin siblings who were eligible and met recruitment criteria were invited to participate as well (Fan et al., 2023). Four of the 21 sites were twin hubs with decades of successful experience in ascertaining and recruiting twins (i.e., the University of Colorado—Boulder, the University of Minnesota—Twin Cities, Virginia Commonwealth University, and Washington University). These four sites recruited twins through direct contact using parental name and contact information from birth registries and rigorous tracking of parents and twins to their current residential address location (Iacono et al., 2018). To reduce potential twin-non-twin confounding by site, each twin hub ascertained twin pairs along with singletons following the same school-based procedures as the rest of the ABCD study (Iacono et al., 2018).

After excluding singletons to focus on twin and non-twin siblings in line with the purpose of the present study, we drew on data from later waves of the ABCD study. Specifically, the ABCD study's 5.1 data release included survey responses from adolescents and their primary caregivers from 2016 (i.e., Wave 1) to 2023 (i.e., Wave 5). The present study used data from Waves 3 (2019–2021) and 4 (2020–2022), when key constructs were available. The University of Minnesota's Institutional Review Board exempted the present study from review due to the analysis of de-identified and publicly available data.

### Measures

#### Psychopathology symptoms

At Waves 3 and 4, adolescents completed the Brief Problem Monitor-Youth Form (BPM-Y; Achenbach, 2009). The online supplement reports our rationale for excluding parent- and teacher-reports of youth's psychopathology symptoms. The BPM-Y asked youth to report their internalizing symptoms (six-item; e.g., I am unhappy, sad, or depressed; α-range_wave_ = .73–.74), externalizing symptoms (seven-item; e.g., *I destroy things belonging to others*; α-range_wave_ = .67–.68), and attention problems (six-item; e.g., *I have trouble concentrating or paying attention*; α-range_wave_ = .76–.77) with three-point Likert scales (0 = *not true*, 2 = *very true*). A confirmatory factor analysis (CFA) indicated that a three-indicator latent variable at each wave fit the data well, *χ*^2^(6) = 45.57, *p* < .001, RMSEA = .07, CFI = .98, SRMR = .03, and constraining the factor loadings to be equivalent across time did not result in a significant decrement in model fit, *Δχ^2^* (3) = 4.27, *p* = .23, enabling us to achieve metric invariance across time for psychopathology symptoms. Notably, assessing the three indicators of psychopathology symptoms as independent yet interrelated outcomes did not change the pattern of our results (see Table S1). Thus, adolescents’ psychopathology symptoms were modeled as a single latent variable at each wave and coded so that high values indicated more psychopathology symptoms.

#### Racial-ethnic discrimination

At Wave 3, adolescents completed a seven-item measure derived from the *Measure of Perceived Discrimination* (Phinney et al., 1998). Across the seven items, adolescents used five-point Likert scales (1 = *never*, 5 = *very often*) to report their racial-ethnic discrimination perceptions from specific and non-specific perpetrators (e.g., *How often do the following people treat you unfairly or negatively because of your ethnic background-Teachers*? *I feel that others behave in an unfair or negative way toward my ethnic groups*?). The measure showed acceptable internal consistency (*α* = .75). Thus, we measured racial-ethnic discrimination as a mean score with higher values indicating more frequent racial-ethnic discrimination perceptions.

#### Racial-ethnic identity development

At Wave 4, adolescents completed the *Multigroup Ethnic Identity Measure* (MEIM; Phinney & Ong, 2007), which included subscales of racial-ethnic identity exploration and commitment. Each subscale included three items. The identity exploration subscale included questions about the extent to which adolescents questioned and sought information about their race-ethnicity (e.g., *I have often talked to other people in order to learn more about my ethnic group*.), and the commitment subscale asked questions about the extent to which adolescents felt a sense of belonging with and commitment to their racial-ethnic group (e.g., *I have a strong sense of belonging to my own ethnic group*.). Each subscale used five-point Likert scales (1 = *strongly disagree*, 5 = *strongly agree*) and showed acceptable internal consistency (*α*_exploration_ = .87; *α*_commitment_ = .88). A series of CFAs illustrated that a two-factor structure fit the data well, *χ*^2^ (8) = 32.79, *p* < .001, RMSEA = .05, CFI = .99, TLI = .98, SRMR = .02, and better than a one-factor structure, *Δχ^2^* (1) = 165.04, *p* < .001. Thus, we distinguished between adolescents’ identity exploration and commitment, wherein each dimension was a mean score with high values indicating greater engagement in identity exploration or commitment.

#### Covariates

As detailed in the online supplement, adolescent-level covariates included youth's Wave-3 psychopathology symptoms, race-ethnicity, sex, and chronological age. Family-level covariates included parental education, parental income, parents’ marital status, and census-tract measures on the percentage of residents living below the poverty line and racial-ethnic diversity. Census-tract racial-ethnic diversity was estimated using the Simpson Diversity Index (Simpson, 1949), which was adapted to account for the relative proportion of each racial-ethnic group and the number of racial-ethnic groups represented within a census tract. The resulting diversity score was a continuous probability score ranging from 0 to 1 with high scores reflecting greater racial-ethnic diversity within a census tract (Benner & Graham, 2009; Graham, 2016). In addition, to specify that our results were attributable to the co-sibling control design, family-level covariates included family-level aggregated measures of discrimination, exploration, and commitment.

### Missing data

Of the 1,184 youth, 1,181 youth participated at Wave 3, and 1,156 youth participated at Wave 4. Across waves, 1,153 youth participated in both waves, and 31 youth participated in only one wave. These two groups of youth did not significantly differ by race-ethnicity, sex, parental education, parental income, parental marital status, census-tract percentage of residents living below the poverty line, and racial-ethnic diversity (see detailed attrition analyses in the online supplement). However, youth who participated in both waves were older than those who participated in only one wave. For our key constructs, no significant differences emerged between both groups of youth on racial-ethnic discrimination, racial-ethnic identity exploration, racial-ethnic identity commitment, and Wave-4 internalizing symptoms, externalizing symptoms, and attention problems. These missing data patterns suggested that our data were conditionally missing at random (Enders, 2023), enabling us to use full information maximum likelihood estimation to retain all 1,184 adolescents with any available data.

### Analytic plan

In *Mplus v. 8.11* (Muthén & Muthén, 1998), we estimated two multilevel models given the hierarchical structure of 1,184 adolescents nested in 656 families across 21 sites. Family cluster sizes of two or more children are sufficient for sibling fixed-effect models (Sjölander et al., 2022). To quantify how much variance in each construct was attributable to each level of the data structure, we estimated a three-level unconditional model with adolescents (Level 1) nested within families (Level 2), who were nested within sites (Level 3). Intraclass correlation coefficients (ICCs) in Table S2 indicated that 72%–82% of the variance was at the individual adolescent level within families (ICC_individual_-range = .72–.82), 15%–27% was shared between siblings in the same family (ICC_family_-range = .15–.27), and 0%–3% was attributable to differences between sites (ICC_site_-range = .00–.03). These ICC values suggested meaningful within-family differences and justified the use of multilevel modeling to account for the nested structure of the data, enabling us to assign Adolescents to Level 1 and Families to Level 2, while using TYPE = COMPLEX to account for site-level random effects. With categorical variables as the exception, Level-1 predictors were family-mean centered to capture relative differences between siblings in the same family, and Level-2 predictors were sample-mean centered to account for between-family differences. To answer our first research question, the Level-1 portion of the first model examined the sibling fixed effect of racial-ethnic discrimination and each racial-ethnic identity component on psychopathology symptoms, controlling for our covariates. Next, for our second research question, we estimated the product of mean-centered terms for racial-ethnic discrimination and each racial-ethnic identity component at each level and, thereafter, included these interaction terms into our equations.

## Results

Table 1 presents descriptive statistics for our key study constructs. Of the 1,184 youth, 449 youth reported at least one instance of discrimination. Between families, 526 youth were in families in which neither sibling reported an instance of discrimination, 480 youth were in households in which one sibling reported an instance of discrimination but the other(s) did not, and 178 youth were in families in which all siblings reported an instance of discrimination (see the online supplement for sensitivity analyses comparing these three groups of youth).

**Table 1:** Means (standard deviations) and zero-order bivariate correlations among key study constructs.

	Key study constructs	1	2	3	4	5	6	7	8	9	Mean (SD)
1	Wave-3 internalizing symptoms	1									0.29 (0.54)
2	Wave-4 internalizing symptoms	.53**	1								0.31 (0.37)
3	Wave-3 externalizing symptoms	.44**	.27**	1							0.32 (0.31)
4	Wave-4 externalizing symptoms	.30**	.40**	.63**	1						0.32 (0.31)
5	Wave-3 attention problems	.50**	.31**	.58**	.43**	1					0.57 (0.46)
6	Wave-4 attention problems	.41**	.51**	.41**	.54**	.64**	1				0.58 (0.46)
7	Wave-3 racial-ethnic discrimination	.25**	.16**	.23**	.19**	.18**	.16**	1			1.22 (0.43)
8	Wave-4 racial-ethnic identity exploration	−.03	−.02	−.01	−.04	−.04	−.07*	.13**	1		3.27 (0.84)
9	Wave-4 racial-ethnic identity commitment	−.03	−.03	−.05	−.07*	−.01	−.05	.06*	.63**	1	3.62 (0.79)

Note.*p < .05, **p < .01.

Table S3 presents zero-order bivariate correlations between our covariates and key study constructs. On average, racial-ethnic identity exploration was associated with lower parental education (*r* = −.12, *p* < .01), less parental income (*r* = −.15, *p* < .15), a lower likelihood of living in a two-parent households (*r* = −.09, *p* < .01), and a greater proportion of residents living below the poverty line in their census tracts (*r* = .06, *p* < .05). Thus, a quasi-experimental design, like a co-sibling control study, adjusting for shared environmental confounds is warranted.

### The main effects of racial-ethnic discrimination on psychopathology symptoms

The left side of Table 2 presents a multilevel model examining the main effects of racial-ethnic discrimination and each racial-ethnic identity development component on psychopathology symptoms one year later, net of our covariates. At Level 1, adolescents perceiving more racial-ethnic discrimination than their siblings reported more psychopathology symptoms one year later [*B* = 0.06, SE = 0.03, 95% CI (0.01, 0.12), *p* < .001]. The model fit the data well, *χ*^2^(63) = 157.65, *p* < .001, RMSEA = .04, CFI = .96, SRMR_within_ = .04, SRMR_between_ = .04.

**Table 2:** Multilevel models examining the main and interactive effects of racial-ethnic discrimination and racial-ethnic identity development on psychopathology symptoms among 1,184 adolescents nested in 656 families.

	Main effects model		Interactive effects model	
Within-family fixed effects	B (SE)	95% CI	B (SE)	95% CI
Black (vs. Latino) youth	−0.09 (0.04)*	[−0.16, −0.02]	−0.09 (0.04)*	[−0.16, −0.02]
Asian (vs. Latino) youth	−0.04 (0.07)	[−0.17, 0.09]	−0.05 (0.08)	[−0.21, 0.11]
Other (vs. Latino) youth	−0.01 (0.04)	[−0.09, 0.06]	0.01 (0.05)	[−0.08, 0.10]
Male (vs. female) youth	−0.04 (0.03)	[−0.09, 0.00]	−0.02 (0.03)	[−0.08, 0.05]
Youth's chronological age	0.01 (0.02)	[−0.03, 0.04]	−0.02 (0.03)	[−0.08, 0.05]
Prior year's psychopathology symptoms	0.62 (0.06)***	[0.50, 0.74]	0.69 (0.05)***	[0.59, 0.79]
Racial-ethnic discrimination	0.06 (0.03)*	[0.01, 0.12]	0.07 (0.04)	[−0.01, 0.15]
REI exploration	−0.02 (0.02)	[−0.06, 0.03]	−0.01 (0.02)	[−0.05, 0.03]
REI commitment	0.02 (0.03)	[−0.04, 0.08]	0.03 (0.03)	[−0.03, 0.09]
Racial-ethnic discrimination × REI exploration	—	—	−0.39 (0.10)***	[−0.58, −0.20]
Racial-ethnic discrimination × REI commitment	—	—	0.12 (0.14)	[−0.16, 0.39]
*R^2^*	0.28		0.33	
Between-family fixed effects				
Parental education	−0.01 (0.01)	[−0.02, 0.00]	−0.01 (0.01)	[−0.02, 0.00]
Parental income	0.00 (0.01)	[−0.02, 0.01]	−0.01 (0.01)	[−0.02, 0.01]
Two (vs. single) parent household	−0.05 (0.04)	[−0.12, 0.02]	−0.05 (0.03)	[−0.11, 0.02]
Census tract—SES	0.00 (0.00)	[0.00, 0.00]	0.00 (0.00)	[0.00, 0.00]
Census tract—racial-ethnic diversity	0.00 (0.08)	[−0.17, 0.16]	−0.07 (0.07)	[−0.21, 0.07]
Racial-ethnic discrimination	0.24 (0.06)***	[0.13, 0.35]	0.26 (0.05)***	[0.16, 0.37]
REI exploration	−0.02 (0.02)	[−0.06, 0.03]	−0.04 (0.02)*	[−0.07, −0.01]
REI commitment	−0.07 (0.03)*	[−0.14, −0.01]	−0.06 (0.03)	[−0.13, 0.01]
Racial-ethnic discrimination × REI exploration	—	—	0.00 (0.08)	[−0.16, 0.16]
Racial-ethnic discrimination × REI commitment	—	—	−0.04 (0.09)	[−0.21, 0.13]
*R^2^*	0.12		0.15	
Intercepts	0.00 (0.00)	[0.00, 0.00]	0.00 (0.00)	[0.00, 0.00]
Residual variance—Level-1 outcome	0.05 (0.01)***	[0.03, 0.06]	0.07 (0.01)***	[0.05, 0.08]
Residual variance—Level-2 outcome	0.07 (0.01)***	[0.05, 0.09]	0.06 (0.01)***	[0.04, 0.08]

*Note.* *p < .05, ***p < .001.

REI = racial-ethnic identity; SES = socioeconomic status.

### The moderating role of racial-ethnic identity development

The right portion of Table 2 examines the main and interactive effects of racial-ethnic discrimination and each racial-ethnic identity component on psychopathology symptoms one year later, after we adjusted for our covariates. When predicting psychopathology symptoms at Level 1, racial-ethnic discrimination significantly interacted with racial-ethnic identity exploration [*B* = −0.39, SE = 0.10, 95% CI (−0.58, −0.20), *p* < .001] but not with racial-ethnic identity commitment [*B* = 0.12, SE = 0.14, 95% CI (−0.16, 0.39), *p* = .40]. Racial-ethnic discrimination did not significantly interact with either racial-ethnic identity component to predict psychopathology symptoms at Level 2, illustrating that the resilience linked to racial-ethnic identity exploration might have been masked by unmeasured between-family differences. The model fit the data well, *χ*^2^(76) = 177.79, *p* < .001, RMSEA = .04, CFI = .94, SRMR_within_ = .05, SRMR_between_ = .05.

**Figure 1:**
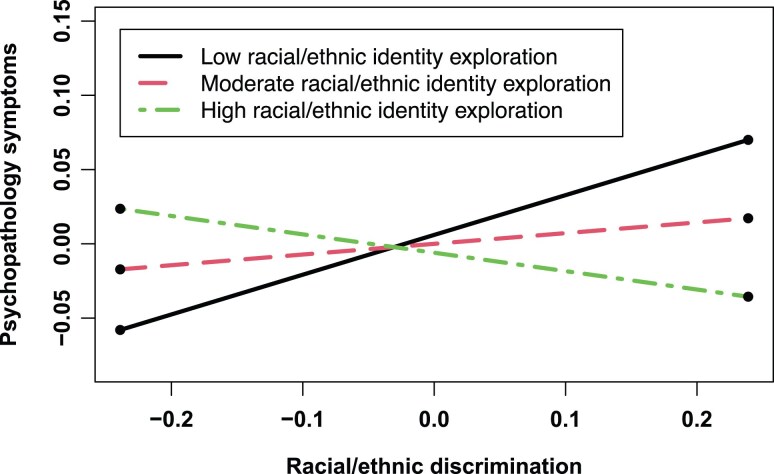
Associations between racial-ethnic discrimination and psychopathology symptoms one year later at 16th (low), 50th (moderate), and 84th (high) percentiles of racial-ethnic identity exploration at the within-family level. The slope for low racial-ethnic identity exploration was significant (B = 0.27, SE = 0.06, 95% CI [0.15, 0.38], *p* < .001), whereas slopes for moderate and high racial-ethnic identity exploration were non-significant [moderate: B = 0.07, SE = 0.04, 95% CI (−0.01, 0.15), *p* = .06; high: B = −0.12, SE = 0.06, 95% CI (−0.25, 0.01), *p* = .06].

To interpret the interactive effect of racial-ethnic discrimination and racial-ethnic identity exploration, Figure 1plots a simple slope analysis at 16th, 50th, and 84th percentiles of .racial-ethnic identity exploration. A significant and positive association between racial-ethnic discrimination and psychopathology symptoms one year later emerged among adolescents at the 16th percentile of racial-ethnic identity exploration [*B* = 0.27, *SE* = 0.06, 95% CI (0.15, 0.38), *p* < .001]; a non-significant positive association between racial-ethnic discrimination and psychopathology symptoms one year later emerged among adolescents at the 50th percentile of racial-ethnic identity exploration [*B* = 0.07, SE = 0.04, 95% CI (−0.01, 0.15), *p* = .06]; and a non-significant inverse association between racial-ethnic discrimination and psychopathology symptoms one year later emerged among adolescents at the 84th percentile of racial-ethnic identity exploration [*B* = −0.12, SE = 0.06, 95% CI (−0.25, 0.01), *p* = .06].

### Sensitivity analyses

We conducted several robustness checks to optimally understand the contours of our results. First, we tested whether our results replicated when we combined racial-ethnic identity exploration and commitment into a single composite score, which is one approach prior work has done (Yip et al., 2019). This combined score produced similar results as racial-ethnic identity exploration (see Table S4), and this result using the combined score may be attributable to the high correlation between racial-ethnic identity exploration and commitment (*r* = .63, *p* < .001). Given this high correlation, we tested whether our results stayed the same after we estimated separate models for each racial-ethnic identity ­component to rule out potential multicollinearity. These results produced similar findings as those in our main analysis (see Tables S5 and S6).

Second, because monozygotic twin pairs are 100% genetically identical and dizygotic twins and full siblings share 50% of their genetic architecture, we leveraged this variability in siblings’ genetic relatedness to test whether our results differed between 216 monozygotic twins versus 938 dizygotic twins and full siblings to rule out the idea that the resilience linked to racial-ethnic identity exploration may be a heritable trait. In doing so, we used siblings’ zygosity as a grouping variable for a multi-group analysis and tested whether we could constrain the main and interactive effects of racial-ethnic discrimination and racial-ethnic identity exploration on psychopathology symptoms to be identical between monozygotic twins versus dizygotic twins and full siblings without causing a significant decrement in model fit. Ultimately, we found that we could without causing a significant decrement in model fit, *χ^2^* (3) = 4.84, *p* = .18, implicating that the protection associated with racial-ethnic identity exploration was not a heritable trait.

Third, although we found that racial-ethnic discrimination did not significantly interact with neither racial-ethnic identity component at Level 2, we examined the interactive of effects racial-ethnic discrimination and each racial-ethnic identity development component on psychopathology symptoms among 2,823 unrelated youth of color to rule out the possibility that our analysis at Level 2 might have been underpowered. In Table S7, racial-ethnic discrimination continued to non-significantly interact with each racial-ethnic identity development component to predict psychopathology symptoms, which is additional evidence that the resilience linked to racial-ethnic identity exploration might be masked by unmeasured between-person differences.

Last, we assessed whether the adaptation linked to racial-­ethnic identity exploration might be invariant across racial-­ethnic groups and between males and females. We shifted youth's race-ethnicity to a grouping variable for multi-group analyses and found that constraining the main and interactive effects of racial-ethnic discrimination and identity exploration on psychopathology symptoms to be equivalent across groups caused a significant decrement in model fit, *χ^2^* (9) = 81.13, *p* < .001, with Latino youth exhibiting the least protection linked to racial-ethnic identity exploration. To examine the moderating role of youth's sex, we distinguished between all-female pairs versus all-male pairs and used this categorical distinction as a grouping variable for multi-group analyses. We found that we could constrain the main and interactive effects of racial-ethnic discrimination and identity exploration on psychopathology symptoms to be equivalent between males and females without causing a significant decrement in model fit, *χ^2^* (3) = 0.77, *p* = .86, suggesting that our main results did not vary by youth's sex.

## Discussion

Racial-ethnic identity development has the potential to protect early adolescents of color against the pernicious effects of racial-ethnic discrimination on their psychological well-being (Benner et al., 2022; Yip, 2018). Of the two components of ­racial-ethnic identity development, the literature has found that racial-ethnic identity commitment offers protection, whereas racial-ethnic identity exploration exacerbates the effects of discrimination on adolescents’ adjustment outcomes (Yip, 2018; Yip et al., 2019). However, most studies examining the protection linked to racial-ethnic identity development have relied on between-person comparisons, cross-sectional data, different age groups, and concurrent assessments of racial-ethnic identity development and discrimination. To address these limitations, we used a longitudinal co-sibling control design with a national sample of early adolescents of color. With this design, we tested whether youth who perceived more racial-ethnic discrimination than their siblings subsequently reported greater psychopathology symptoms one year later. Next, we examined whether this longitudinal association between discrimination and psychopathology symptoms was moderated by sibling discordant scores on racial-ethnic identity development, measured at the follow-up year.

Consistent with our first hypothesis, early adolescents who perceived more frequent racial-ethnic discrimination than their siblings reported more psychopathology symptoms one year later. This initial finding supports the PVEST (Spencer et al., 1997), which describes how the interpretations derived from racial-ethnic discrimination experiences become internalized, potentially reinforcing maladaptive stress responses and heightening youth's psychopathology symptoms (Cunningham et al., 2023; Spencer, 1995). This process may be especially the case in early adolescence, a developmental period when youth are uniquely attuned to their social environments (Del Toro et al., 2025b). In addition, in our supplemental analyses, youth in households in which all siblings perceived at least one instance of discrimination also reported high scores on indicators of socioeconomic disadvantage. This finding may indicate that the types of discrimination youth are perceiving are possibly correlated with structural consequences of racism (e.g., racialized economic inequities). These supplemental analyses also illustrated that the group of youth in households in which a single youth perceived discrimination did not significantly differ on socio-demographic factors relative to youth in households in which neither sibling perceived discrimination, suggesting that the findings derived from the former group of youth may generalize to the latter group once a sibling perceives discrimination. Altogether, our findings corroborated previous empirical studies and meta-analyses that found racial-ethnic discrimination was robustly associated with greater psychopathology symptoms (Benner et al., 2022; Del Toro & Wang, 2023; Wang et al., 2025).

In line with our second hypothesis, racial-ethnic identity exploration conferred early adolescents with protection against racial-ethnic discrimination. Youth reporting more racial-ethnic identity exploration than their siblings one year following their perceived discrimination exhibited weaker longitudinal associations between racial-ethnic discrimination and psychopathology symptoms. In line with the PVEST (Cunningham et al., 2023; Spencer, 2006), the reactive coping aspect of racial-ethnic identity exploration measured following racial-ethnic discrimination may have enabled early adolescents of color to engage in meaning-making processes that incorporate amenable adaptive responses to discrimination. These youth with high levels of racial-ethnic identity exploration may have achieved a moratorium identity status, characterized by active exploration of their race-ethnicity without a strong commitment to it (Marcia, 1994), which may have allowed youth to flexibly adapt their identities across various social contexts. This cognitive maturity and self-reflection may have enabled youth to consider discrimination as one of many ­experiences rather than a defining one (McLean & Pasupathi, 2012). In addition, the developmental appropriateness for early adolescents to explore and ask questions about their race-ethnicity may have facilitated the resilience linked to identity exploration, as prior research illustrated that racial-ethnic identity exploration was more likely to exacerbate the effects of racial-ethnic discrimination on adjustment outcomes in adulthood, a period when exploration may be discouraged due to societal pressures for individuals to enter the workforce, pursue post-graduate education, and establish intimate partnerships (Arnett, 2000; Yip et al., 2019). These societal expectations may push older youth to commit rather than explore their identities.

To our surprise, racial-ethnic identity commitment did not moderate the association between racial-ethnic discrimination and psychopathology symptoms. The lack of a significant moderation effect for racial-ethnic identity commitment may be attributable to racial-ethnic identity commitment conferring protection for some youth but not for other youth. For example, some youth may have benefited from a sense of belonging and commitment to their racial-ethnic group (Yip et al., 2019), while other youth may have felt hurt that discrimination targeted an identity that they felt committed to (e.g., Cheeks et al., 2020). These two opposing psycho-social processes, if true, may have cancelled each other out and contributed to a non-significant moderation effect for racial-ethnic identity commitment. Another possible reason racial-ethnic identity commitment did not buffer the effects of discrimination on psychopathology symptoms may relate to the temporal nature of both racial-ethnic identity commitment and psychopathology symptoms. Specifically, racial-ethnic identity commitment is relatively stable across time and context (Del Toro et al., 2023; Del Toro et al., 2024a), whereas psychopathology symptoms often fluctuate considerably across days or even within a single day (Del Toro & Wang, 2023; Wright & Simms, 2016). When a stable individual difference is used to moderate a dynamic outcome, particularly one that is influenced by recent stressors, the misalignment in their temporal properties may mask potential buffering effects. Supporting this notion, a recent meta-analysis found that racial-ethnic identity commitment was protective for academic, cognitive, and physical health, but not for mental health outcomes (Yip et al., 2019). This finding suggests that the utility of racial-ethnic identity commitment as a protective factor may depend, in part, on the temporal nature of the outcome it is expected to influence.

Building on prior studies, we used a novel approach and applied a co-sibling control study that disaggregated between unshared versus shared environments between siblings in the same family. A focus on unshared environments between siblings provided us with a closer snapshot of adolescents’ phenomenological experiences, as we compared adolescents’ resilience relative to that of their siblings. This comparison adjusted for shared environmental factors, including confounds associated with racial-ethnic identity exploration. Recall, in our online supplement, youth with high levels of racial-ethnic identity exploration were, on average, likely to live with parents who have lower incomes, less advanced educational degrees, single marital statuses, and live in census tracts with greater proportions of residents living below the poverty line. These contextual factors may have made it hard for researchers to disaggregate youth's phenomenological experiences from their environments in prior between-person comparisons. When we conducted similar between-person comparisons among the singleton sample, we found a non-significant moderation effect for racial-ethnic identity exploration, which is additional evidence that between-person comparisons can induce biased estimates. With intertwined associations between race-ethnicity and socioeconomic disadvantage, it can be unclear whether an adolescent's vulnerability to psychopathology is attributable to their identity development or exposures to social inequities. By accounting for between-person confounds, a co-sibling control study is one optimal way to capture theory-driven processes about youth's coping and adaptation.

### Limitations and directions for future research

The present study's limitations warrant consideration. First, the racial-ethnic identity development measure was assessed one year following the racial-ethnic discrimination measure, when traditional approaches in the field assessed both racial-ethnic identity development and discrimination at the same time (Yip et al., 2019). However, the fact that racial-ethnic identity development was measured after racial-ethnic discrimination positioned identity as a reactive coping method, in line with the PVEST (Spencer et al., 1997). Nonetheless, whether our results replicate when both discrimination and identity are assessed concurrently is a helpful direction for future research. Second, while sibling fixed-effect models help account for unobserved family-level confounds, they do not fully address all individual-level factors that vary within families, such as youth's unique interactions with their environments. Third, the generalizability of the findings may be limited, as our approach excluded only children and those without sibling data. This exclusion could have introduced selectin bias, but the similarity of results when analyzing the singleton sample and the between-family portion of our models suggest that any bias from this exclusion is likely minimal. Fourth, racial-ethnic identity development reflects one dimension of identity (Ashmore et al., 2004), as there are other components of identity (e.g., content models; Sellers et al., 1997) that were not measured and, ultimately, not included in the present study. Fifth, the racial-ethnic groups in our study reflected pan-ethnic labels, providing directions for future research to consider within-group heterogeneity (Volpe et al., 2022).

### Implications

The current study has implications for both practice and theory. Practically, our findings indicate that efforts to support racial-ethnic identity exploration may help buffer youth against the psychological harms of racial-ethnic discrimination. Although our analyses relied on sibling discordant scores, this approach is conceptually similar to other multilevel frameworks where adolescents are nested within classrooms or schools (Burt, 2014). Just as studies comparing classmates or schoolmates remain generalizable, within-family comparisons strengthen causal inference by accounting for unmeasured genetic and shared environmental confounders (Rutter, 2007). In this way, our results provide stronger confidence about the protective role of racial-ethnic identity exploration at the individual level. In other words, even within the same family environment, adolescents who explored their racial-ethnic identities were better protected against ­discrimination than their siblings who explored less. By attempting to closely isolate the unique protective process linked to racial-ethnic identity exploration, our study complements prior intervention work showing that programs, which encourage youth to learn about and reflect on their racial-ethnic backgrounds promote resilience (Bonilla et al., 2021; Dee & Penner, 2017; Umaña-Taylor et al., 2018). Families can support youth by encouraging open conversations about their racial-ethnic background and sharing stories of cultural pride and history (Stein et al., 2021). Clinicians and program developers can offer guided activities like cultural journaling, interviews with elders, and heritage discussions, without pressuring youth to prematurely settle on a fixed identity. Indeed, the current study points to racial-ethnic identity exploration as a promising and practical target for interventions designed to support youth facing discrimination.

For theory, this study challenges an implicit assumption that youth may not benefit and may even be at risk when they engage in racial-ethnic identity exploration. Much of this assumption stems from cross-sectional studies, which may have conflated family-level influences with individual-level identity processes. Our findings showed that racial-ethnic identity exploration did not appear protective in between-family analyses or among singletons but did confer protection in sibling-comparison analyses that adjusted for shared familial confounds. This quasi-experimental evidence suggests that earlier conclusions about exploration being neutral or harmful may be partly spurious. Theoretical models of identity development should therefore reconsider negative assumptions about exploration and explicitly account for shared environmental influences that can mask its protective properties.

## Conclusion

The present study contributes to a large body of research on how early adolescents of color navigate racial-ethnic discrimination and its effects on psychopathology symptoms through the lens of racial-ethnic identity development. Using a co-sibling control design, which minimized shared environmental confounds and provided more precise estimates than typical between-person comparisons, we found that youth who perceived more discrimination than their siblings reported more psychopathology symptoms one year later than their siblings. Critically, we also demonstrated that racial-ethnic identity exploration buffered these adolescents against the negative effects of discrimination on psychopathology symptoms, providing novel evidence for its protection, which has been inconsistently supported in the extant literature (e.g., Yip et al., 2019). Relative to prior studies’ negative depiction of racial-ethnic identity exploration, the present study found that this process of exploring one's racial-ethnic identity is a key developmental asset that fosters adolescents’ resilience in the face of racial-ethnic discrimination. These insights have direct implications for youth programs and interventions, underscoring the importance of creating safe, affirming spaces for identity exploration. In a world where discrimination continues to shape the daily lives of youth of color, supporting racial-ethnic identity exploration is a necessary act of care, resistance, and repair.

## Supplementary Material

aacaf048_Supplementary_Data

## Data Availability

The data necessary to reproduce the analyses presented here are publicly available, and the analytic code and the materials necessary to reproduce the analyses in the present manuscript are not publicly available but are available from the first author upon reasonable request. The analyses presented here were not preregistered. Data from the present study came from the 5.1 release of the Adolescent Brain Cognitive Development (ABCD) Study (http://dx.doi.org/10.15154/z563-zd24). ABCD consortium investigators designed and implemented the study and provided data but did not participate in the analysis or writing of this report. This manuscript reflects the views of the authors and may not reflect the opinions or views of the NIH or ABCD consortium investigators.
