# Supplementary material for: The explorer's edge: Racial-ethnic identity exploration confers early adolescents of color with protection against racial-ethnic discrimination in a co-sibling control study
Source: Child Dev. 2026 Feb 11;97(2):556–69. doi: 10.1093/chidev/aacaf048 (PMC13046085; doi:10.1093/chidev/aacaf048)
Supplement: aacaf048_Supplementary_Data [file aacaf048_supplementary_data.docx]

**Supplemental Information**

**Rationale: Excluding Parent- and Teacher-Reports of Youth’s Psychopathology Symptoms**

At Waves 3 and 4, teachers and parents also reported on adolescents’ psychopathology symptoms, using the Brief Problem Monitor-Teacher Form (Achenbach, 2009) and the Child Behavior Checklist (Achenbach et al., 2016), respectively. However, teacher-reports at Waves 3 and 4 were missing for 67% and 82% of the analytic sample, respectively, and teachers have been found to view racial-ethnic minoritized youth’s behaviors as problematic for subjective and non-violent infractions (Del Toro & Wang, 2023b). Furthermore, psychopathology symptoms are inherently subjective and often not easily observable by others (De Los Reyes et al., 2015), and racial-ethnic minoritized parents may under-report youth’s symptoms due to cultural expectations and limited visibility into key contexts where symptoms manifest (e.g., peer settings, schools; Yeh & Weisz, 2001), which was supported by our data as we found that parents under-reported youth’s symptoms for two of the three outcomes at Waves 3 and 4, including externalizing symptoms [Wave 3: *t(*890) = 18.21, *p* < .001; Wave 4: *t(*873) = 17.97, *p* < .001] and attention problems [Wave 3: *t(*890) = 23.45, *p* < .001; Wave 4: *t(*872) = 24.02, *p* < .001]. Altogether, given the present study’s emphasis on youth’s phenomenology, our primary analyses were restricted to youth’s subjective, self-reports of their psychopathology symptoms.

**Covariates**

Adolescent-level covariates included youth’s Wave-3 psychopathology symptoms, race-ethnicity (i.e., Black: 0 = *Latino*, 1 = *Black*; Asian: 0 = *Latino*, 1 = *Asian*; Other youth of color: 0 = *Latino*, 1 = *Other youth of color*), sex (0 = *female*, 1 = *female*), and chronological age (range = 11-12). Family-level covariates included parental education (0 = *never attended/kindergarten only*, 21 = *doctoral degree*), parental income (1 = *less than $5,000*; 10 = *$200,000 and greater*), parents’ marital status (0 = *widowed, divorced, separated, or never married*, 1 = *married or living partner*), and census-tract measures on the percentage of residents living below poverty (range = 0-1; 0 = *0%*, 1 = *100%*) and racial-ethnic diversity. Census-tract racial-ethnic diversity was estimated using the Simpson Diversity Index (Simpson, 1949), which was adapted to account for the relative proportion of each racial-ethnic group and the number of racial-ethnic groups represented within a census tract. The resulting diversity score was a continuous probability score ranging from 0 to 1 with high scores reflecting greater racial-ethnic diversity within a census tract (Benner & Graham, 2009; Graham, 2016). In addition, to specify that our results were attributable to the co-sibling control design, family-level covariates included family-level aggregated measures of discrimination, exploration, and commitment.

**Missing Data**

Of the 1,184 adolescents, 1,181 youth participated at Wave 3, and 1,156 youth participated at Wave 4. Across waves, 1,153 youth participated in both waves, and 31 youth participated in only one wave. These two groups of youth did not significantly differ by race-ethnicity [*χ^2^* (3) = 1.86, *p* = .60], sex [*χ^2^* (1) = 0.99, *p* = .32], parental education [*t*(1183) = 0.12, *p* = .90], parental income [*t*(50.10) = 0.55, *p* = .59], parental marital status [*χ^2^* (1) = 3.03, *p* = .08], census-tract percentage of residents living below poverty [*t*(47.42) = 1.84, *p* = .07], and racial-ethnic diversity [*t*(1120) = 1.73, *p* = .08]. However, youth who participated in both waves were older than those who participated in only one wave [*t*(31.97) = 2.59, *p* < .05]. For our key constructs, no significant differences emerged between both groups of youth on racial-ethnic discrimination [*t*(1158) = 0.54, *p* = .59], racial-ethnic identity exploration [*t*(1185) = 0.04, *p* = .97], racial-ethnic identity commitment [*t*(1185) = 0.39, *p* = .70], and Wave-4 internalizing symptoms [*t*(1181) = 0.15, *p* = .88], externalizing symptoms [*t*(1182) = 0.50, *p* = .62], and attention problems [*t*(1182) = 0.27, *p* = .79]. These missing data patterns suggested that our data were conditionally missing at random (Enders, 2023), enabling us to use full information maximum likelihood estimation to retain all 1,184 adolescents with any available data.

**Descriptive and Inferential Statistics Comparing the Three Groups of Adolescents with Varying Exposures to Racial-ethnic Discrimination within the Household**

Our descriptive statistics raised three questions. First, what statistical information could the 526 youth provide when not a single adolescent in the household has experienced racial-ethnic discrimination? – The sample of 526 youth who were in households in which neither sibling experienced discrimination will not contribute statistically meaningful information at the within-family level but can contribute meaningful information to between-family comparisons, enabling us to compare the resilience linked to racial-ethnic identity components across within- and between-family levels. Nonetheless, excluding the sample of 526 youth produced a similar pattern of results as our main analysis (see Tables S8).

Second, the sub-sample of 480 adolescents, in households in which one adolescent experienced discrimination whereas the other siblings did not, raises a question about whether the presence versus absence of discrimination of discrimination, in addition to its frequency (e.g., more versus less exposure to discrimination), matters for adolescent development. Among this sub-sample of 480 adolescents, we tested whether discrimination dichotomized (0 = *unexposed to discrimination*, 1 = *exposed to discrimination*) produced similar results as our main analysis. In these analyses, the interactive effect of dichotomized racial-ethnic discrimination and racial-ethnic identity exploration on psychopathology symptoms remained significant (see Table S9), elucidating that both the presence and relative frequency of discrimination are meaningful for adolescent development.

The third question that this sub-sample of 480 adolescent raises is whether such adolescents are driving our main results and whether they may generalize to other samples. After we restricted our analysis to the 178 adolescents whose siblings were all exposed to discrimination at least once, we found that the interactive effect of the frequency of racial-ethnic discrimination and racial-ethnic identity exploration remained significant (see Table S10). Nonetheless, to contextualize the differences among the three sub-samples, Table S11 presents comparisons of the three groups of youth nested in households with siblings exposed to varying levels of discrimination. Youth in households in which all siblings experienced discrimination were more likely to be Black, less likely to be Latino, and less likely to live in two-parent households than youth in households in which neither sibling experienced discrimination. On average, relative to youth in households in which no sibling experienced discrimination, youth in households in which all siblings experienced discrimination were more likely to be Black and less likely to be Latino; less likely to live in two-parent households; more likely to live in households with lower parental income; and more likely to live in more racially/ethnically diverse census tracts.

| **Table S1**  *Multilevel Models Examining the Interactive Effects of Racial-Ethnic Discrimination and Racial-Ethnic Identity Development on Each Indicator of Psychopathology Symptoms among 1,184 Adolescents Nested in 656* *Families* | | | | | | | | |
| --- | --- | --- | --- | --- | --- | --- | --- | --- |
|  | Attention problems | |  | Internalizing symptoms | |  | Externalizing symptoms | |
| Within-family fixed effects | *B (SE)* | *95% CI* | | *B (SE)* | *95% CI* | | *B (SE)* | *95% CI* |
| Black (vs. Latino) youth | -0.07 (0.03)* | [-0.13, -0.01] | | -0.06 (0.04) | [-0.12, 0.01] | | -0.05 (0.03) | [-0.12, 0.00] |
| Asian (vs. Latino) youth | -0.05 (0.09) | [-0.22, 0.11] | | -0.08 (0.03)* | [-0.15, -0.02] | | 0.02 (0.05) | [-0.08, 0.12] |
| Other (vs. Latino) youth | 0.01 (0.04) | [-0.07, 0.10] | | -0.01 (0.03) | [-0.08, 0.06] | | 0.00 (0.03) | [-0.05, 0.07] |
| Male (vs. female) youth | 0.00 (0.03) | [-0.05, 0.05] | | -0.12 (0.02)*** | [-0.17, -0.07] | | -0.01 (0.01) | [-0.04, 0.02] |
| Youth’s chronological age | -0.04 (0.04) | [-0.12, 0.03] | | -0.01 (0.04) | [-0.08, 0.06] | | 0.04 (0.03) | [-0.01, 0.08] |
| Prior year’s psychopathology symptoms | 0.53 (0.03)*** | [0.48, 0.58] | | 0.39 (0.06)*** | [0.28, 0.51] | | 0.56 (0.03)*** | [0.50, 0.61] |
| Racial-ethnic discrimination | 0.09 (0.04)* | [0.01, 0.17] | | 0.11 (0.04)** | [0.04, 0.18] | | 0.04 (0.04) | [-0.04, 0.12] |
| REI exploration | -0.02 (0.02) | [-0.06, 0.03] | | 0.01 (0.02) | [-0.03, 0.04] | | -0.01 (0.02) | [-0.05, 0.03] |
| REI commitment | 0.02 (0.03) | [-0.03, 0.08] | | 0.02 (0.02) | [-0.02, 0.06] | | 0.02 (0.03) | [-0.03, 0.07] |
| Racial-ethnic discrimination x REI exploration | -0.41 (0.11)*** | [-0.62, -0.20] | | -0.22 (0.07)** | [-0.35, -0.08] | | -0.22 (0.07)** | [-0.36, -0.08] |
| Racial-ethnic discrimination x REI commitment | 0.08 (0.15) | [-0.22, 0.37] | | 0.09 (0.11) | [-0.12, 0.30] | | 0.11 (0.14) | [-0.16, 0.38] |
| *R^2^* | 0.20 |  | | 0.13 |  | | 0.20 |  |
| Between-family fixed effects |  |  | |  |  | |  |  |
| Parental education | -0.01 (0.01) | [-0.02, 0.01] | | 0.00 (0.00) | [-0.01, 0.01] | | -0.01 (0.01) | [-0.01, 0.01] |
| Parental income | -0.01 (0.01) | [-0.02, 0.01] | | 0.00 (0.01) | [-0.02, 0.01] | | -0.01 (0.01) | [-0.02, 0.01] |
| Two (vs. single) parent household | -0.04 (0.03) | [-0.10, 0.03] | | 0.02 (0.02) | [-0.02, 0.07] | | -0.06 (0.03)* | [-0.11, -0.01] |
| Census tract – SES | 0.00 (0.00) | [0.00, 0.00] | | 0.00 (0.00) | [0.00, 0.00] | | 0.00 (0.00) | [0.00, 0.00] |
| Census tract – Racial-ethnic diversity | 0.00 (0.08) | [-0.16, 0.15] | | -0.11 (0.06) | [-0.22, 0.01] | | -0.06 (0.05) | [-0.16, 0.03] |
| Racial-ethnic discrimination | 0.22 (0.06)*** | [0.11, 0.34] | | 0.20 (0.04)*** | [0.12, 0.27] | | 0.18 (0.05)** | [0.08, 0.28] |
| REI exploration | -0.05 (0.02)* | [-0.09, -0.01] | | -0.01 (0.02) | [-0.06, 0.03] | | -0.01 (0.02) | [-0.06, 0.03] |
| REI commitment | -0.04 (0.04) | [-0.12, 0.03] | | -0.06 (0.02)* | [-0.10, -0.01] | | -0.04 (0.03) | [-0.10, 0.01] |
| Racial-ethnic discrimination x REI exploration | 0.03 (0.09) | [-0.14, 0.20] | | 0.05 (0.06) | [-0.07, 0.17] | | -0.05 (0.05) | [-0.15, 0.04] |
| Racial-ethnic discrimination x REI commitment | -0.05 (0.09) | [-0.23, 0.13] | | -0.09 (0.08) | [-0.25, 0.07] | | 0.02 (0.06) | [-0.11, 0.15] |
| *R^2^* | 0.09 |  | | 0.14 |  | | 0.18 |  |
| Intercepts | 0.64 (0.03)*** | [0.57, 0.71] | | 0.39 (0.04)*** | [0.31, 0.47] | | 0.39 (0.03)*** | [0.33, 0.44] |
| Residual variance – Level-1 outcome | 0.10 (0.01)*** | [0.09, 0.12] | | 0.09 (0.01)*** | [0.08, 0.11] | | 0.05 (0.01)*** | [0.04, 0.06] |
| Residual variance – Level-2 outcome | 0.08 (0.01)*** | [0.06, 0.10] | | 0.03 (0.00)*** | [0.02, 0.04] | | 0.03 (0.00)*** | [0.02, 0.03] |
| *Note*. * *p* < .05, ** *p* < .01, *** *p* < .001. *REI = Racial-ethnic identity*; *SES = Socioeconomic status* | | | | | | | | |

| **Table S2**  *Intraclass Correlation Coefficients for Key Study Constructs* | | | |
| --- | --- | --- | --- |
| Key study constructs | Adolescents within families | Families within study sites | Between study sites |
| Wave-4 internalizing symptoms | .81 | .18 | .01 |
| Wave-4 externalizing symptoms | .81 | .19 | .00 |
| Wave-4 attention problems | .73 | .25 | .02 |
| Wave-3 racial-ethnic discrimination | .72 | .27 | .01 |
| Wave-4 racial-ethnic identity exploration | .79 | .19 | .02 |
| Wave-4 racial-ethnic identity commitment | .82 | .15 | .03 |

| **Table S3**  *Zero-Order Bivariate Correlations Between Covariates and Key Study Constructs* | | | | | | | | | |
| --- | --- | --- | --- | --- | --- | --- | --- | --- | --- |
| Covariates | Wave-3 internalizing symptoms | Wave-4 internalizing symptoms | Wave-3 externalizing symptoms | Wave-4 externalizing symptoms | Wave-3 attention problems | Wave-4 attention problems | Wave-3 racial-ethnic discrimination | Wave-4  REI exploration | Wave-4  REI commitment |
| Black (vs. non-Black) youth | -.02 | -.06* | .00 | -.04 | .00 | -.05 | .16** | .17** | .20** |
| Asian (vs. non-Asian) youth | -.03 | -.03 | -.04 | -.03 | -.06 | -.04 | -.05 | -.05 | -.05 |
| Other (vs. non-Other) youth | .02 | .02 | -.01 | .00 | -.01 | .02 | -.09** | -.08** | -.11** |
| Latino (vs. non-Latino) youth | .01 | .05 | .03 | .05 | .03 | .05 | -.06* | -.08** | -.09** |
| Male (vs. female) youth | -.10** | -.18** | .00 | -.01 | .07* | -.02 | .04 | -.02 | -.03 |
| Youth’s chronological age | -.03 | -.02 | -.01 | -.02 | .01 | -.02 | .01 | -.01 | -.03 |
| Parental education | .01 | .01 | -.02 | -.05 | -.05 | -.02 | -.06 | -.12** | -.13** |
| Parental income | -.03 | .00 | -.06 | -.08* | -.06* | -.04 | -.12** | -.15** | -.09** |
| Two (vs. single) parent household | -.02 | .03 | -.10** | -.09** | -.09** | -.02 | -.09** | -.09** | -.04 |
| Census tract – SES | .02 | .00 | .04 | .01 | .00 | .00 | .12** | .06* | .08** |
| Census tract – Racial-ethnic diversity | .02 | -.02 | .01 | .00 | .01 | .02 | .06* | .00 | -.04 |
| *Note*. * *p* < .05, ** *p* < .01. *REI = Racial-ethnic identity; SES = Socioeconomic status.* | | | | | | | | | |

| **Table S4**  *Multilevel Models Examining the Main and Interactive Effects of Racial-Ethnic Discrimination and a Single Composite Score for Racial-Ethnic Identity Development on Psychopathology Symptoms among 1,184 Adolescents in 656* *Families* | | | | | |
| --- | --- | --- | --- | --- | --- |
|  | Main effects model | |  | Interactive effects model | |
| Within-family fixed effects | *B (SE)* | *95% CI* | | *B (SE)* | *95% CI* |
| Black (vs. Latino) youth | -0.11 (0.04)** | [-0.19, -0.04] | | -0.11 (0.04)** | [-0.18, -0.03] |
| Asian (vs. Latino) youth | -0.03 (0.08) | [-0.18, 0.12] | | -0.03 (0.08) | [-0.18, 0.12] |
| Other (vs. Latino) youth | -0.01 (0.05) | [-0.10, 0.08] | | -0.01 (0.04) | [-0.10, 0.07] |
| Male (vs. female) youth | -0.03 (0.03) | [-0.09, 0.02] | | -0.03 (0.03) | [-0.09, 0.03] |
| Youth’s chronological age | -0.01 (0.03) | [-0.07, 0.05] | | -0.01 (0.03) | [-0.06, 0.05] |
| Prior year’s psychopathology symptoms | 0.63 (0.06)*** | [0.51, 0.75] | | 0.62 (0.06)*** | [0.51, 0.74] |
| Racial-ethnic discrimination | 0.07 (0.03)* | [0.01, 0.14] | | 0.08 (0.03)* | [0.01, 0.15] |
| REI single composite score | 0.01 (0.02) | [-0.03, 0.05] | | 0.01 (0.02) | [-0.03, 0.06] |
| Racial-ethnic discrimination x REI single composite score | -- | -- | | -0.27 (0.11)** | [-0.49, -0.06] |
| *R^2^* | 0.27 |  | | 0.28 |  |
| Between-family fixed effects |  |  | |  |  |
| Parental education | -0.01 (0.01) | [-0.02, 0.00] | | -0.01 (0.01) | [-0.02, 0.00] |
| Parental income | -0.01 (0.01) | [-0.02, 0.01] | | -0.01 (0.01) | [-0.02, 0.01] |
| Two (vs. single) parent household | -0.05 (0.03) | [-0.11, 0.02] | | -0.04 (0.03) | [-0.12, 0.02] |
| Census tract – SES | 0.00 (0.00) | [0.00, 0.00] | | 0.00 (0.00) | [0.00, 0.00] |
| Census tract – Racial-ethnic diversity | -0.06 (0.08) | [-0.21, 0.09] | | -0.07 (0.07) | [-0.22, 0.06] |
| Racial-ethnic discrimination | 0.32 (0.04)*** | [0.25, 0.40] | | 0.31 (0.04)*** | [0.23, 0.39] |
| REI single composite score | -0.10 (0.02)*** | [-0.14, -0.06] | | -0.10 (0.02)*** | [-0.14, -0.06] |
| Racial-ethnic discrimination x REI single composite score | -- | -- | | -0.02 (0.10) | [-0.21, 0.17] |
| *R^2^* | 0.17 |  | | 0.16 |  |
| Intercepts | 0.00 (0.00) | [0.00, 0.00] | | 0.00 (0.00) | [0.00, 0.00] |
| Residual variance – Level-1 outcome | 0.05 (0.01)*** | [0.04, 0.07] | | 0.07 (0.01)*** | [0.05, 0.08] |
| Residual variance – Level-2 outcome | 0.06 (0.01)*** | [0.04, 0.08] | | 0.06 (0.01)*** | [0.04, 0.07] |
| *Note*. * *p* < .05, ** *p* < .01, *** *p* < .001. *REI = Racial-ethnic identity*; *SES = Socioeconomic status.* | | | | | |

| **Table S5**  *Multilevel Models Examining the Main and Interactive Effects of Racial-Ethnic Discrimination and Racial-Ethnic Identity Exploration on Psychopathology Symptoms among 1,184 Adolescents in 656* *Families* | | | | | |
| --- | --- | --- | --- | --- | --- |
|  | Main effects model | |  | Interactive effects model | |
| Within-family fixed effects | *B (SE)* | *95% CI* | | *B (SE)* | *95% CI* |
| Black (vs. Latino) youth | -0.10 (0.04)** | [-0.18, -0.03] | | -0.10 (0.04)** | [-0.18, -0.03] |
| Asian (vs. Latino) youth | -0.05 (0.07) | [-0.18, 0.09] | | -0.05 (0.08) | [-0.21, 0.11] |
| Other (vs. Latino) youth | -0.01 (0.04) | [-0.09, 0.06] | | 0.01 (0.04) | [-0.07, 0.10] |
| Male (vs. female) youth | -0.04 (0.03) | [-0.09, 0.01] | | -0.01 (0.03) | [-0.08, 0.05] |
| Youth’s chronological age | 0.00 (0.02) | [-0.03, 0.04] | | -0.02 (0.03) | [-0.08, 0.05] |
| Prior year’s psychopathology symptoms | 0.62 (0.06)*** | [0.50, 0.74] | | 0.69 (0.05)*** | [0.60, 0.79] |
| Racial-ethnic discrimination | 0.06 (0.03)* | [0.01, 0.12] | | 0.07 (0.04) | [-0.01, 0.15] |
| REI exploration | 0.00 (0.02) | [-0.03, 0.03] | | 0.00 (0.02) | [-0.03, 0.03] |
| Racial-ethnic discrimination x REI exploration | -- | -- | | -0.34 (0.08)*** | [-0.49, -0.19] |
| *R^2^* | 0.29 |  | | 0.33 |  |
| Between-family fixed effects |  |  | |  |  |
| Parental education | -0.01 (0.01) | [-0.02, 0.01] | | -0.01 (0.01) | [-0.02, 0.01] |
| Parental income | 0.00 (0.01) | [-0.02, 0.01] | | -0.01 (0.01) | [-0.02, 0.01] |
| Two (vs. single) parent household | -0.06 (0.04) | [-0.13, 0.01] | | -0.05 (0.03) | [-0.12, 0.01] |
| Census tract – SES | 0.00 (0.00) | [-0.01, 0.01] | | 0.00 (0.00) | [-0.01, 0.01] |
| Census tract – Racial-ethnic diversity | 0.01 (0.09) | [-0.16, 0.18] | | -0.05 (0.08) | [-0.20, 0.10] |
| Racial-ethnic discrimination | 0.24 (0.06)*** | [0.13, 0.36] | | 0.24 (0.06)*** | [0.12, 0.37] |
| REI exploration | -0.06 (0.01)*** | [-0.08, -0.04] | | -0.07 (0.01)*** | [-0.09, -0.05] |
| Racial-ethnic discrimination x REI exploration | -- | -- | | 0.00 (0.09) | [-0.17, 0.17] |
| *R^2^* | 0.10 |  | | 0.13 |  |
| Intercepts | 0.00 (0.00) | [0.00, 0.00] | | 0.00 (0.00) | [0.00, 0.00] |
| Residual variance – Level-1 outcome | 0.05 (0.01)*** | [0.03, 0.06] | | 0.06 (0.01)*** | [0.05, 0.08] |
| Residual variance – Level-2 outcome | 0.08 (0.01)*** | [[0.05, 0.10] | | 0.06 (0.01)*** | [0.04, 0.08] |
| *Note*. * *p* < .05, ** *p* < .01, *** *p* < .001. *REI = Racial-ethnic identity*; *SES = Socioeconomic status* | | | | | |

| **Table S6**  *Multilevel Models Examining the Main and Interactive Effects of Racial-Ethnic Discrimination and Racial-Ethnic Identity Commitment on Psychopathology Symptoms among 1,184 Adolescents in 656* *Families* | | | | | |
| --- | --- | --- | --- | --- | --- |
|  | Main effects model | |  | Interactive effects model | |
| Within-family fixed effects | *B (SE)* | *95% CI* | | *B (SE)* | *95% CI* |
| Black (vs. Latino) youth | -0.09 (0.04)* | [-0.17, -0.02] | | -0.10 (0.04)** | [-0.17, -0.03] |
| Asian (vs. Latino) youth | -0.04 (0.07) | [-0.17, 0.09] | | -0.05 (0.08) | [-0.21, 0.10] |
| Other (vs. Latino) youth | -0.01 (0.04) | [-0.09, 0.07] | | 0.01 (0.05) | [-0.08, 0.10] |
| Male (vs. female) youth | -0.04 (0.03) | [-0.09, 0.01] | | -0.02 (0.03) | [-0.09, 0.05] |
| Youth’s chronological age | 0.00 (0.02) | [-0.04, 0.04] | | -0.02 (0.03) | [-0.09, 0.05] |
| Prior year’s psychopathology symptoms | 0.62 (0.06)*** | [0.50, 0.74] | | 0.71 (0.05)*** | [0.61, 0.81] |
| Racial-ethnic discrimination | 0.06 (0.03)* | [0.01, 0.12] | | 0.06 (0.04) | [-0.01, 0.14] |
| REI commitment | 0.01 (0.02) | [-0.03, 0.06] | | 0.02 (0.02) | [-0.03, 0.07] |
| Racial-ethnic discrimination x REI commitment | -- | -- | | -0.09 (0.15) | [-0.39, 0.21] |
| *R^2^* | 0.29 |  | | 0.32 |  |
| Between-family fixed effects |  |  | |  |  |
| Parental education | -0.01 (0.01) | [-0.02, 0.01] | | -0.01 (0.01) | [-0.02, 0.01] |
| Parental income | 0.00 (0.01) | [-0.02, 0.01] | | -0.01 (0.01) | [-0.02, 0.01] |
| Two (vs. single) parent household | -0.05 (0.03) | [-0.12, 0.02] | | -0.04 (0.03) | [-0.11, 0.02] |
| Census tract – SES | 0.00 (0.00) | [-0.01, 0.01] | | 0.00 (0.00) | [-0.01, 0.01] |
| Census tract – Racial-ethnic diversity | -0.01 (0.08) | [-0.17, 0.15] | | -0.06 (0.07) | [-0.21, 0.08] |
| Racial-ethnic discrimination | 0.24 (0.05)*** | [0.13, 0.35] | | 0.24 (0.05)*** | [0.14, 0.34] |
| REI commitment | -0.08 (0.02)*** | [-0.12, -0.04] | | -0.08 (0.03)** | [-0.13, -0.03] |
| Racial-ethnic discrimination x REI commitment | -- | -- | | -0.03 (0.09) | [-0.20, 0.14] |
| *R^2^* | 0.12 |  | | 0.14 |  |
| Intercepts |  |  | |  |  |
| Residual variance – Level-1 outcome | 0.05 (0.01)*** | [0.03, 0.06] | | 0.07 (0.01)*** | [0.05, 0.08] |
| Residual variance – Level-2 outcome | 0.07 (0.01)*** | [0.05, 0.09] | | 0.06 (0.01)*** | [0.04, 0.08] |
| *Note*. * *p* < .05, ** *p* < .01, *** *p* < .001. *REI = Racial-ethnic identity*; *SES = Socioeconomic status* | | | | | |

| **Table S7**  *Multilevel Models Examining the Main and Interactive Effects of Racial-ethnic Discrimination and Racial-Ethnic Identity Development on Psychopathology Symptoms among 2,823 Singleton Adolescents* | | | | | |
| --- | --- | --- | --- | --- | --- |
|  | Main effects model | |  | Interactive effects model | |
| Within-family fixed effects | *B (SE)* | *95% CI* | | *B (SE)* | *95% CI* |
| Black (vs. Latino) youth | -0.03 (0.02) | [-0.07, 0.00] | | -0.03 (0.02) | [-0.07, 0.00] |
| Asian (vs. Latino) youth | -0.07 (0.03)* | [-0.13, -0.01] | | -0.07 (0.03)* | [-0.14, -0.01] |
| Other (vs. Latino) youth | 0.01 (0.02) | [-0.04, 0.05] | | 0.00 (0.02) | [-0.04, 0.05] |
| Male (vs. female) youth | -0.11 (0.01)*** | [-0.13, -0.09] | | -0.11 (0.01)*** | [-0.13, -0.09] |
| Youth’s chronological age | 0.01 (0.01) | [-0.01, 0.03] | | 0.01 (0.01) | [-0.01, 0.03] |
| Parental education | 0.00 (0.00) | [-0.01, 0.01] | | 0.00 (0.00) | [-0.01, 0.01] |
| Parental income | 0.00 (0.00) | [-0.01, 0.01] | | 0.00 (0.00) | [-0.01, 0.01] |
| Two (vs. single) parent household | 0.02 (0.02) | [-0.02, 0.06] | | 0.02 (0.02) | [-0.02, 0.06] |
| Census tract – SES | 0.00 (0.00) | [0.00, 0.00] | | 0.00 (0.00) | [0.00, 0.00] |
| Census tract – Racial-ethnic diversity | 0.08 (0.04)* | [0.01, 0.16] | | 0.08 (0.04)* | [0.01, 0.16] |
| Prior year’s psychopathology symptoms | 0.68 (0.03)*** | [0.63, 0.73] | | 0.68 (0.02)*** | [0.63, 0.73] |
| Racial-ethnic discrimination | 0.04 (0.02)* | [0.01, 0.06] | | 0.04 (0.02)* | [0.01, 0.08] |
| REI exploration | -0.01 (0.01) | [-0.03, 0.01] | | -0.01 (0.01) | [-0.03, 0.01] |
| REI commitment | -0.01 (0.01) | [-0.04, 0.01] | | -0.01 (0.01) | [-0.04, 0.02] |
| Racial-ethnic discrimination x REI exploration | -- | -- | | -0.02 (0.02) | [-0.06, 0.02] |
| Racial-ethnic discrimination x REI commitment | -- | -- | | 0.01 (0.03) | [-0.05, 0.07] |
| *R^2^* | 0.47 |  | | 0.47 |  |
| Intercepts | 0.00 (0.00) | [0.00, 0.00] | | 0.00 (0.00) | [0.00, 0.00] |
| Residual variance – outcome | 0.07 (0.01)*** | [0.06, 0.08] | | 0.07 (0.01)*** | [0.06, 0.08] |
| *Note*. * *p* < .05, ** *p* < .01, *** *p* < .001. *REI = Racial-ethnic identity*; *SES = Socioeconomic status.* | | | | | |

| **Table S8**  *Multilevel Models Examining the Main and Interactive Effects of Racial-Ethnic Discrimination and Racial-Ethnic Identity Development on Psychopathology Symptoms among 658 Adolescents Nested in 318* *Families* | | | | | |
| --- | --- | --- | --- | --- | --- |
|  | Main effects model | |  | Interactive effects model | |
| Within-family fixed effects | *B (SE)* | *95% CI* | | *B (SE)* | *95% CI* |
| Black (vs. Latino) youth | -0.05 (0.04) | [-0.14, 0.03] | | -0.01 (0.03) | [-0.08, 0.05] |
| Asian (vs. Latino) youth | 0.00 (0.06) | [-0.12, 0.13] | | 0.01 (0.09) | [-0.17, 0.19] |
| Other (vs. Latino) youth | -0.04 (0.04) | [-0.13, 0.04] | | -0.01 (0.04) | [-0.09, 0.08] |
| Male (vs. female) youth | -0.08 (0.03)** | [-0.13, -0.02] | | -0.07 (0.04) | [-0.14, 0.00] |
| Youth’s chronological age | 0.00 (0.02) | [-0.04, 0.05] | | -0.03 (0.04) | [-0.10, 0.04] |
| Prior year’s psychopathology symptoms | 0.58 (0.06)*** | [0.45, 0.70] | | 0.62 (0.07)*** | [0.49, 0.75] |
| Racial-ethnic discrimination | 0.07 (0.03)* | [0.01, 0.13] | | 0.09 (0.04)* | [0.02, 0.16] |
| REI exploration | -0.03 (0.03) | [-0.09, 0.03] | | -0.02 (0.03) | [-0.08, 0.04] |
| REI commitment | 0.03 (0.03) | [-0.02, 0.08] | | 0.03 (0.02) | [-0.01, 0.08] |
| Racial-ethnic discrimination x REI exploration | -- | -- | | -0.39 (0.09)*** | [-0.54, -0.22] |
| Racial-ethnic discrimination x REI commitment | -- | -- | | 0.09 (0.12) | [-0.14, 0.33] |
| *R^2^* | 0.31 |  | | 0.35 |  |
| Between-family fixed effects |  |  | |  |  |
| Parental education | -0.01 (0.01) | [-0.03, 0.01] | | -0.01 (0.01) | [-0.03, 0.01] |
| Parental income | 0.01 (0.01) | [-0.02, 0.03] | | 0.00 (0.01) | [-0.02, 0.03] |
| Two (vs. single) parent household | -0.05 (0.05) | [-0.15, 0.05] | | -0.06 (0.06) | [-0.17, 0.05] |
| Census tract – SES | 0.00 (0.00) | [-0.01, 0.00] | | 0.00 (0.00) | [-0.01, 0.00] |
| Census tract – Racial-ethnic diversity | -0.09 (0.09) | [-0.27, 0.08] | | -0.15 (0.07)* | [-0.29, -0.01] |
| Racial-ethnic discrimination | 0.18 (0.06)** | [0.06, 0.30] | | 0.22 (0.06)*** | [0.10, 0.34] |
| REI exploration | -0.01 (0.03) | [-0.07, 0.06] | | -0.03 (0.03) | [-0.08, 0.02] |
| REI commitment | -0.08 (0.04)* | [-0.15, -0.01] | | -0.07 (0.04)* | [-0.15, -0.01] |
| Racial-ethnic discrimination x REI exploration | -- | -- | | 0.02 (0.07) | [-0.12, 0.16] |
| Racial-ethnic discrimination x REI commitment | -- | -- | | -0.08 (0.12) | [-0.30, 0.15] |
| *R^2^* | 0.08 |  | | 0.13 |  |
| Intercepts | 0.00 (0.00) | [0.00, 0.00] | | 0.00 (0.00) | [0.00, 0.00] |
| Residual variance – Level-1 outcome | 0.04 (0.01)*** | [0.03, 0.05] | | 0.06 (0.01)*** | [0.04, 0.08] |
| Residual variance – Level-2 outcome | 0.08 (0.02)*** | [0.04, 0.13] | | 0.06 (0.02)** | [0.03, 0.10] |
| *Note*. * *p* < .05, ** *p* < .01, *** *p* < .001. *REI = Racial-ethnic identity*; *SES = Socioeconomic status* | | | | | |

| **Table S9**  *Multilevel Models Examining the Main and Interactive Effects of Racial-ethnic Discrimination and Racial-Ethnic Identity Development on Psychopathology Symptoms among 480 Adolescents Nested in 228* *Families* | | | | | |
| --- | --- | --- | --- | --- | --- |
|  | Main effects model | |  | Interactive effects model | |
| Within-family fixed effects | *B (SE)* | *95% CI* | | *B (SE)* | *95% CI* |
| Black (vs. Latino) youth | -0.04 (0.06) | [-0.15, 0.07] | | -0.03 (0.05) | [-0.13, 0.08] |
| Asian (vs. Latino) youth | -0.13 (0.13) | [-0.39, 0.13] | | -0.10 (0.13) | [-0.36, 0.15] |
| Other (vs. Latino) youth | -0.03 (0.05) | [-0.13, 0.08] | | -0.01 (0.05) | [-0.11, 0.09] |
| Male (vs. female) youth | -0.06 (0.04) | [-0.15, 0.03] | | -0.05 (0.04) | [-0.14, 0.04] |
| Youth’s chronological age | -0.06 (0.05) | [-0.16, 0.04] | | -0.05 (0.05) | [-0.15, 0.04] |
| Prior year’s psychopathology symptoms | 0.57 (0.10)*** | [0.38, 0.76] | | 0.58 (0.09)*** | [0.39, 0.76] |
| Racial-ethnic discrimination | 0.04 (0.04) | [-0.03, 0.12] | | 0.04 (0.04) | [-0.03, 0.11] |
| REI exploration | -0.03 (0.04) | [-0.11, 0.05] | | 0.08 (0.06) | [-0.04, 0.20] |
| REI commitment | -0.01 (0.03) | [-0.06, 0.04] | | 0.00 (0.06) | [-0.11, 0.12] |
| Racial-ethnic discrimination x REI exploration | -- | -- | | -0.21 (0.10)* | [-0.40, -0.01] |
| Racial-ethnic discrimination x REI commitment | -- | -- | | -0.03 (0.10) | [-0.23, 0.17] |
| *R^2^* | 0.27 |  | | 0.29 |  |
| Between-family fixed effects |  |  | |  |  |
| Parental education | -0.01 (0.01) | [-0.04, 0.02] | | -0.01 (0.01) | [-0.04, 0.02] |
| Parental income | 0.00 (0.02) | [-0.04, 0.04] | | 0.00 (0.02) | [-0.04, 0.03] |
| Two (vs. single) parent household | -0.05 (0.09) | [-0.22, 0.12] | | -0.04 (0.08) | [-0.20, 0.13] |
| Census tract – SES | 0.00 (0.00) | [-0.01, 0.00] | | 0.00 (0.00) | [-0.01, 0.00] |
| Census tract – Racial-ethnic diversity | -0.07 (0.07) | [-0.21, 0.07] | | -0.10 (0.07) | [-0.23, 0.04] |
| Racial-ethnic discrimination | -- | -- | | -- | -- |
| REI exploration | -0.03 (0.05) | [-0.12, 0.07] | | -0.02 (0.05) | [-0.12, 0.07] |
| REI commitment | -0.07 (0.04) | [-0.16, 0.01] | | -0.09 (0.04)* | [-0.17, -0.01] |
| Racial-ethnic discrimination x REI exploration | -- | -- | | -- | -- |
| Racial-ethnic discrimination x REI commitment | -- | -- | | -- | -- |
| *R^2^* | 0.06 |  | | 0.07 |  |
| Intercepts | 0.00 (0.00) | [0.00, 0.00] | | 0.00 (0.00) | [0.00, 0.00] |
| Residual variance – Level-1 outcome | 0.06 (0.01)*** | [0.03, 0.10] | | 0.06 (0.01)*** | [0.04, 0.09] |
| Residual variance – Level-2 outcome | 0.09 (0.02)*** | [0.04, 0.15] | | 0.09 (0.02)*** | [0.04, 0.13] |
| *Note*. * *p* < .05, ** *p* < .01, *** *p* < .001. *REI = Racial-ethnic identity*; *SES = Socioeconomic status* | | | | | |

| **Table S10**  *Multilevel Models Examining the Main and Interactive Effects of Racial-Ethnic Discrimination and Racial-Ethnic Identity Development on Psychopathology Symptoms among 178 Adolescents Nested in 88* *Families* | | | | | |
| --- | --- | --- | --- | --- | --- |
|  | Main effects model | |  | Interactive effects model | |
| Within-family fixed effects | *B (SE)* | *95% CI* | | *B (SE)* | *95% CI* |
| Black (vs. Latino) youth | -0.08 (0.09) | [-0.25, 0.08] | | -0.06 (0.09) | [-0.23, 0.10] |
| Asian (vs. Latino) youth | 0.00 (0.05) | [-0.10, 0.10] | | 0.03 (0.08) | [-0.13, 0.19] |
| Other (vs. Latino) youth | -0.03 (0.06) | [-0.15, 0.09] | | -0.04 (0.06) | [-0.16, 0.08] |
| Male (vs. female) youth | -0.06 (0.05) | [-0.17, 0.04] | | -0.05 (0.06) | [-0.17, 0.06] |
| Youth’s chronological age | 0.01 (0.04) | [-0.07, 0.09] | | 0.01 (0.04) | [-0.07, 0.09] |
| Prior year’s psychopathology symptoms | 0.76 (0.14)*** | [0.48, 1.04] | | 0.72 (0.14)*** | [0.44, 0.99] |
| Racial-ethnic discrimination | 0.04 (0.06) | [-0.08, 0.16] | | 0.07 (0.07) | [-0.07, 0.20] |
| REI exploration | 0.01 (0.05) | [-0.09, 0.11] | | 0.03 (0.05) | [-0.07, 0.13] |
| REI commitment | 0.08 (0.06) | [-0.04, 0.21] | | 0.07 (0.06) | [-0.05, 0.19] |
| Racial-ethnic discrimination x REI exploration | -- | -- | | -0.44 (0.15)** | [-0.74, -0.14] |
| Racial-ethnic discrimination x REI commitment | -- | -- | | 0.25 (0.24) | [-0.22, 0.71] |
| *R^2^* | 0.58 |  | | 0.63 |  |
| Between-family fixed effects |  |  | |  |  |
| Parental education | -0.01 (0.02) | [-0.06, 0.03] | | -0.01 (0.02) | [-0.04, 0.02] |
| Parental income | 0.00 (0.03) | [-0.05, 0.05] | | 0.00 (0.02) | [-0.04, 0.05] |
| Two (vs. single) parent household | 0.00 (0.05) | [-0.10, 0.11] | | -0.01 (0.07) | [-0.14, 0.13] |
| Census tract – SES | 0.00 (0.00) | [-0.01, 0.00] | | 0.00 (0.00) | [-0.01, 0.00] |
| Census tract – Racial-ethnic diversity | -0.17 (0.52) | [-1.19, 0.86] | | -0.18 (0.47) | [-1.10, 0.73] |
| Racial-ethnic discrimination | 0.03 (0.26) | [-0.48, 0.55] | | 0.11 (0.19) | [-0.27, 0.50] |
| REI exploration | -0.05 (0.09) | [-0.21, 0.12] | | -0.08 (0.07) | [-0.22, 0.06] |
| REI commitment | 0.04 (0.11) | [-0.17, 0.25] | | 0.04 (0.09) | [-0.15, 0.22] |
| Racial-ethnic discrimination x REI exploration | -- | -- | | 0.17 (0.12) | [-0.06, 0.39] |
| Racial-ethnic discrimination x REI commitment | -- | -- | | -0.25 (0.26) | [-0.75, 0.25] |
| *R^2^* | 0.07 |  | | 0.28 |  |
| Intercepts | 0.00 (0.00) | [0.00, 0.00] | | 0.00 (0.00) | [0.00, 0.00] |
| Residual variance – Level-1 outcome | 0.03 (0.01)*** | [0.01, 0.04] | | 0.03 (0.01)*** | [0.01, 0.04] |
| Residual variance – Level-2 outcome | 0.05 (0.07) | [-0.10, 0.19] | | 0.04 (0.04) | [-0.03, 0.11] |
| *Note*. * *p* < .05, ** *p* < .01, *** *p* < .001. *REI = Racial-ethnic identity*; *SES = Socioeconomic status* | | | | | |

| **Table S11**  *Demographic Characteristics by Sub-Samples of Adolescents Nested in Households with Variations in Siblings Exposed to Racial-Ethnic Discrimination* | | | | | |
| --- | --- | --- | --- | --- | --- |
| Key study variables | Statistic | No siblings were exposed to discrimination  (*n* = 526) | A single sibling was exposed to discrimination  (*n* = 480) | All siblings were exposed to discrimination  (*n* = 178) | One-way ANOVA or *χ^2^* test |
| Black | % | 26.40 | 36.90 | 55.10 | *χ^2^* (6) = 53.70, *p* < .001 |
| Latinx | % | 39.90 | 37.30 | 23.00 | -- |
| Asian | % | 03.60 | 03.80 | 01.10 | -- |
| Other | % | 30.00 | 22.10 | 20.80 | -- |
| Females | % | 51.00 | 53.30 | 52.20 | *χ^2^* (2) = 0.57, *p* = .75 |
| Males | % | 49.00 | 46.60 | 47.80 | -- |
| Age at baseline | *M (SD)* | 09.51 (0.50) | 09.49 (0.50) | 09.52 (0.52) | *F*(2, 1179) = 0.23, *p* = .79 |
| Two (vs. single) parents | % | 71.60 | 61.80 | 53.40 | *χ^2^* (2) = 22.84, *p* < .001 |
| Parental education | *M (SD)* | 15.51 (3.07) | 15.46 (2.71) | 15.56 (2.66) | *F*(2, 1179) = 0.09, *p* = .91 |
| Parental income | *M (SD)* | 06.67 (2.39)_a_ | 06.39 (2.56)_a, b_ | 06.07 (2.60)_b_ | *F*(2, 1070) = 3.69, *p* < .05 |
| Census tract – SES | *M (SD)* | 15.88 (12.14) | 17.16 (13.26) | 17.99 (15.18) | *F*(2, 1119) = 2.07, *p* = .13 |
| Census tract – Racial-ethnic diversity | *M (SD)* | 00.42 (0.18)_b_ | 00.42 (0.18)_b_ | 00.46 (0.17)_a_ | *F*(2, 1119) = 3.74, *p* < .05 |
| *Note*. Different subscripts connote significant group differences at *p* < .05. *SES = Socioeconomic status*. | | | | | |
